# Endocrine rhythms in the brown bear (*Ursus arctos*): Evidence supporting selection for decreased pineal gland size

**DOI:** 10.1002/phy2.48

**Published:** 2013-08-22

**Authors:** Jasmine V Ware, O Lynne Nelson, Charles T Robbins, Patrick A Carter, Brice A J Sarver, Heiko T Jansen

**Affiliations:** 1 Departments of Integrative Physiology and Neuroscience, College of Veterinary Medicine, Washington State UniversityPullman, Washington, 99164; 2 Veterinary Clinical Sciences, College of Veterinary Medicine, Washington State UniversityPullman, Washington, 99164; 3 School of the Environment, Washington State UniversityPullman, Washington, 99164; 4 School of Biological Sciences, Washington State UniversityPullman, Washington, 99164; 5 Department of Biological Sciences, University of IdahoMoscow, Idaho, 83844

**Keywords:** brown bear, cortisol, melatonin, pineal, seasonal

## Abstract

Many temperate zone animals adapt to seasonal changes by altering their physiology. This is mediated in large part by endocrine signals that encode day length and regulate energy balance and metabolism. The objectives of this study were to determine if the daily patterns of two important hormones, melatonin and cortisol, varied with day length in captive brown bears *(Ursus arctos)* under anesthetized and nonanesthetized conditions during the active (March–October) and hibernation periods. Melatonin concentrations varied with time of day and season in nonanesthetized female bears despite exceedingly low nocturnal concentrations (1–4 pg/mL) in the active season. In contrast, melatonin concentrations during hibernation were 7.5-fold greater than those during the summer in anesthetized male bears. Functional assessment of the pineal gland revealed a slight but significant reduction in melatonin following nocturnal light application during hibernation, but no response to beta-adrenergic stimulation was detected in either season. Examination of pineal size in two bear species bears combined with a phylogenetically corrected analysis of pineal glands in 47 other species revealed a strong relationship to brain size. However, pineal gland size of both bear species deviated significantly from the expected pattern. Robust daily plasma cortisol rhythms were observed during the active season but not during hibernation. Cortisol was potently suppressed following injection with a synthetic glucocorticoid. The results suggest that melatonin and cortisol both retain their ability to reflect seasonal changes in day length in brown bears. The exceptionally small pineal gland in bears may be the result of direct or indirect selection.

## Introduction

Photoperiodic time measurement is an important trait that has evolved to enable individuals to reliably predict when to engage in functions such as migration, reproduction, and hibernation (Bradshaw and Holzapfel 2007). Brown bears have evolved a highly seasonal physiology that allows them to effectively acclimatize to various temperate climes, widely changing food availability, and exposure to increasing human influence (Garshelis and Pelton 1980; Gunther 1990; MacHutchon et al. 1998; Olson et al. 1998; Kaczensky et al. 2006; Munro et al. 2006; Nellemann et al. 2007; Schwartz et al. 2010; Ordiz et al. 2011). We have previously shown that captive brown bears use light and food to synchronize (entrain) daily locomotor rhythms in a seasonally dependent manner (Ware et al. 2012). However, the underlying mechanisms used to entrain these rhythms and facilitate the behavioral flexibility remain unknown.

Seasonal rhythms in physiology depend heavily on the interaction between environmental cues and a neuroendocrine system that can be organized in a temporal fashion for entrainment (Bartness et al. 1993; Cauter and Buxton 2001). The product of the pineal gland, melatonin, is secreted into the bloodstream at night and is suppressed by light during the day to create a daily endocrine rhythm (Arendt 1988, 1995). Importantly, the duration of nightly melatonin secretion varies in direct proportion to the length of dark phase in many species, thereby creating an endocrine signature of day length (photoperiod) (Goldman et al. 1984; Arendt 1995; Goldman 2001; Pevet et al. 2006). Sheep have been used as a model for many years to examine the role of the pineal gland and melatonin in mediating seasonal changes in physiology (Malpaux et al. 2001; Hazlerigg et al. 2004). The daily and annual changes in melatonin secretion are mediated by a series of neural pathways beginning in the retina where light:dark information is conveyed via the retinohypothalamic tract to the suprachiasmatic nucleus of the hypothalamus (SCN) (Refinetti 2006). The SCN in turn projects via a multisynaptic pathway to the pineal gland to stimulate melatonin synthesis (Refinetti 2006). Once produced, melatonin binds to G-protein coupled receptors (MT1, MT2) found in a variety of hypothalamic regions (Scherbarth and Steinlechner 2010). In this way, melatonin is thought to modulate adaptations to the annual photoperiodic cycle (Hazlerigg and Wagner 2006; Scherbarth and Steinlechner 2010), but whether or not this is true for bears remains to be determined.

Similar to melatonin, the adrenal hormone cortisol (or corticosterone) exhibits a daily rhythm requiring the SCN (Moore and Eichler 1972; Raisman and Brown-Grant 1977; Kaneko et al. 1981; Buijs et al. 1999). The influence of light on cortisol is also indirect, involving both the hypothalamus and pituitary (Lightman et al. 2002). Cortisol plays an important role in stimulating gluconeogenesis and lipolysis and for the mobilization of amino acids to regulate energy balance (Goodman 2009). The amplitude of the daily cortisol rhythm varies seasonally in many species, including those that exhibit annual body mass and hibernation cycles (Saboureau et al. 1979; Bubenik et al. 1983; Palumbo et al. 1983; Harlow and Beck 1990; Gower et al. 1996). Elevated serum cortisol during hibernation is thought to increase lipolysis necessary to provide energy and maintain the relatively elevated metabolic rate of bears during hibernation (Palumbo et al. 1983; Tøien et al. 2011). The catabolic effects of elevated cortisol appear to be mitigated by circulating binding proteins and shunting of nitrogen products into anabolic pathways during hibernation (Lundberg et al. 1976; Tinker et al. 1998; Chow et al. 2010). The effects of cortisol on energy homeostasis are therefore important mediators of seasonal adaptations (Lemos et al. 2009).

Considering the profound annual changes in body weight (Nelson 1980), cycles of hibernation (Folk et al. 1976), and reproduction (Craighead et al. 1969; Farley and Robbins 1995) exhibited by brown bears, together with their wide geographic distribution in northern latitudes (McLellan et al. 2008), we hypothesized that the daily rhythms of cortisol and melatonin would expand and contract in accordance with changes in day length. Furthermore, we predicted that melatonin and cortisol would be responsive to manipulations shown in other species to enhance or suppress their respective neuroendocrine axes. Lastly, the size of the pineal gland was compared phylogenetically across species to investigate the role of selection on pineal size in the brown bear.

## Methods

### Animals

Male (*n* = 3; wild born, but two raised in captivity since cubs; aged 6–9 years) and female (*n* = 4; captive born; aged 3–8 years during studies) brown bears housed at the Washington State University (WSU) Bear Education, Conservation and Research Center (WSU Bear Center, 46°43′53″ N/117°10′43″ W) were used. Bears were maintained according to the *Bear Care and Colony Health Standard Operating Procedures* with all procedures approved by the Washington State University Institutional Animal Care and Use Committee. Bears were housed in pairs in dens (3 m × 3 m × 2.5 m) with continuous access to an adjacent outdoor run (3 m × 5 m × 5 m). During the active season (March–October), bears were released daily for 6–12 hours into an adjacent 0.56 ha outdoor enclosure. At this time the bears were exposed to natural changes in photoperiod (ranging from 13 hours light: 11 hours dark (L:D) in March/October to 16L:8D in June) and temperature fluctuations (average monthly temperature ranged between 1°C in March and 18°C in July). Bears were fed a commercial diet of dry chow (25.3% protein, 16.2% fat, 51.7% carbohydrate, and 2.0% crude fiber; Hill's Pet Nutrition, Topeka, KS) combined with apples, and small amounts of meat and pastries. Feedings occurred twice daily at 0700 ± 1 hour and 1600 ± 1 hour (standard time). Bears were also allowed to forage for grasses and clover in the irrigated outdoor enclosure to supplement their commercial food diet (Rode et al. 2001). Bears were fed at, or slightly above, maintenance levels between April and August. Then, between August and October, when bears’ appetite increases dramatically (hyperphagia), feeding amounts were increased to well above maintenance levels to provide adequate energy for sufficient fat accumulation necessary to survive the 5 months of hibernation when bears are not eating. In early to mid-October, depending on the bears’ appetite, food amounts were gradually reduced until food was completely withdrawn in late October. Hibernation was considered to have begun when all food was withdrawn (October 24 ± 7 days) or when bears refused food, whichever occurred earliest. Water was available ad libitum. Bears hibernated in the same dens and runs as in the active season with straw provided for bedding. During hibernation the bears were exposed to natural photoperiod (ranging between 11L:13D in February to 8.5L:15:5D at the winter solstice) and temperature fluctuations (average monthly temperature ranged from −1°C in December to 1°C in February). Hibernation ended based on subjective evaluation of general increases in activity at which time feeding was restored (March 1 ± 16 days).

### Endocrine measurements

Blood samples were obtained from four trained female (nonanesthetized) bears between 2008 and 2011 during the Spring (March; 12L:12D), Summer (June; 16L:8D), Late Summer (August; 14L:10D), and Fall (October; 11L:13D). In 2008, two of the four bears were classified as juveniles and reached puberty in 2009 based on reproductive maturation in previous reports (Craighead et al. 1969). These age classifications were not factored into the statistical analyses because of small sample size. Bears were trained using positive reinforcement with food (dilute honey water) to offer their rear limb voluntarily for blood sampling from the dorsal metatarsal or lateral saphenous veins. Blood samples could not be collected from this cohort of bears during hibernation because food is no longer a useful reward given their anorectic state.

To collect blood samples during hibernation (2008–2009 hibernation season: late December to early January; 8L:16D), we sampled three anesthetized adult male bears via jugular venipuncture. Two of these male bears were also anesthetized to collect blood samples during the active season (June; 16L:8D) for direct comparison with hibernation samples. Bears were anesthetized with 1.2 mg/kg of tiletamine HCl and zolazepam HCl (Telazol, Pfizer Animal Health, New York, NY) and 0.08 mg/kg of medetomidine HCl (Dormosedan, Pfizer Animal Health, New York, NY). Following blood sampling, 3–5 mL atipamezole HCl (Antisedan, Pfizer Animal Health, New York, NY) was administered I.V. as a reversal agent. Apexa artificial tears eye lubricant (Bausch and Lomb, Madison, NJ) was placed in the bears’ eyes during anesthesia. Because of the known effects of anesthesia on the hypothalamic–pituitary–adrenal axis, we obtained blood samples no earlier than 45 minutes postanesthesia based on a cortisol decay curve determined in a preliminary study using two adult male bears.

Blood samples were collected from unanesthetized female bears over a 7 ± 5-day period to compile the 24-hours endocrine profiles. Blood samples from all bears were collected into heparinized tubes, centrifuged (1750*g*) for 20 minutes, and the plasma was stored at −80°C until assayed for cortisol or melatonin as described below.

### Radioimmunoassay

Plasma was assayed for melatonin and cortisol by commercially available radioimmunoassay kits (melatonin: ALPCO Diagnostics, Salem, NH; cortisol: MP Biomedicals, Solon, OH, respectively). The assay detection limits were 0.3–0.84 pg/mL for melatonin, depending on lot, and 1.7 ng/mL for cortisol. Both extraction and nonextraction methods were compared in the detection of melatonin in bear plasma according to manufacturer's instructions. Differences between extracted versus nonextracted melatonin concentrations in bear samples were 2% for the low internal control and 15% for the med internal control (high internal control was above assay standard, >81 pg/mL for both extracted and nonextracted assays). In the nonextracted assay, samples were subjected to an enzymatic pretreatment step which removed potentially interfering proteins, followed by acidification and precipitation using a protease mix. The precipitate was removed by centrifugation. Although acceptable ranges for variability were found between assays methods, the extraction method was chosen as the preferred method in all subsequent assays to reduce any potential nonspecific interference. Briefly, 1 mL of plasma was thawed and placed onto a C_18_ reverse-phase column. After passing through the extraction column, the liquid phase was discarded and melatonin was eluted from the column with methanol. The methanol was then evaporated and the remaining melatonin was reconstituted in sample buffer and frozen until assayed. Duplicate 400 μL samples were then incubated with G280 antimelatonin antibody (Vaughan 1993) and ^125^I-melatonin tracer for 20 hours at 4°C. Next, 100 μL of solid-phase bound anti-donkey secondary antibody was added and the tubes were incubated for 15 minutes. One milliliter of double distilled and deionized water was then added and the tubes were centrifuged at 2000*g* in order to precipitate the solid-phase bound antigen:antibody complex. The supernatant was decanted, the tubes were allowed to air dry. Once dry, the precipitates were subjected to gamma counting (Apex 10/880 plus gamma counter, MP Biomedicals, Solon, OH). Extraction efficiency was 99% (determined by spiking a bear daytime sample with ^125^I melatonin and counting pre- and posteluting). Chi-square tests of parallelism for serially diluted samples indicated no difference between expected and measured values. Low, medium, and high internal controls made from pooled daytime bear serum were used to confirm consistent results between assay runs. Intraassay variation was 10.6% and interassay variation was 15.6%.

For cortisol, 25 μL of nonextracted plasma was assayed in duplicate in a competitive binding assay with ^125^I-cortisol tracer in rabbit anticortisol antibody-coated polypropylene tubes. Reagents were incubated for 45 minutes in a water bath at 37 ± 1°C. Liquids were decanted and tubes drained before counting in a gamma counter. Cortisol serial dilution curves confirmed parallelism using the Chi-square test for independence (*P* > 0.05). Intra- and interassay variation was 10.0% and 17.7%, respectively. Cortisol was chosen as the glucocorticoid to measure for two reasons: 1) virtually all previous bear work reported cortisol concentrations and we wanted to make direct comparisons, and 2) cortisol predominates over corticosterone in the brown bear (Koren et al. 2012).

### Pineal gland

Initial examination of adult brown bear necropsy specimens (*n* = 3 males, *n* = 1 female) revealed an extremely small pineal gland for their large brain size compared to other commonly studied seasonal species such as sheep. For a more in-depth examination of the pineal in situ, we also performed a series of MRI studies on postmortem brown bear, polar bear, sheep, and dog brains at the WSU Veterinary Teaching Hospital. These studies used a Phillips 1.0T MRI. Both T1 and T2 modalities in coronal and horizontal sections. Section thickness was 3.5 mm. For within-animal comparisons, the imaged brains were then removed from the heads, fixed in 10% formalin, and analyzed as follows. The width of the pineal gland was measured in mm using a micrometer. Brain width was measured in an identical manner. For both structures, the widest point was recorded. In addition to analysis of bear, sheep, and dog pineal we also made measurements of pineal gland size in 45 other mammalian species (total of 49 species) using an online collection of mammalian brains (http://brainmuseum.org). Similar to our MRI analyses, pineal size was determined using the coronal series of Nissl-stained sections. The section containing the largest pineal was used for analysis. Three measurements were made for each pineal from each species and the average width was used in subsequent analyses. Brain width was analyzed in the same way. Pineal and brain measurements were log transformed and the resulting regression was used to detect outliers by the ROUT method (Motulsky and Brown 2006).

### Phylogenetic analysis

A phylogenetic analysis of pineal sizes among mammalian species was also performed. Most comparative statistical methods assume sample independence. However, simple among-species comparisons violate this assumption because of shared ancestry (Felsenstein 1985). Phylogenetic comparative methods have now been developed to account for shared ancestry; these methods were applied to the data on brain size and pineal size data from the 49 different mammalian species. Specifically, a phylogenetic generalized least squares (PGLS) (Grafen 1989) analysis was used to test for a correlation between brain size and pineal gland size.

A supertree consisting of 4510 mammalian species (Bininda-Emonds et al. 2007) was pruned to include the 49 species for which brain and pineal gland sizes were obtained. A correlation structure was then derived using Pagel's λ, which rescales the tree branch lengths relative to their phylogenetic signal (Pagel 1999; Freckleton et al. 2002; Revell 2010). A linear model (brain size ∼ pineal gland size) was then fitted using generalized least squares. This approach was implemented in the R programming language (R Development Core Team 2012) using the packages *ape* (Paradis et al. 2004; Paradis 2012), *geiger* (Harmon et al. 2008), and *phylobase* (Geiser and Kenagy 1987).

### Neuroendocrine function

Pineal gland responsiveness was determined by: (1) application of bright light to test for melatonin suppression during hibernation, and (2) administration of the beta-adrenergic agonist isoproterenol to test for melatonin stimulation during hibernation and active season. For melatonin suppression, animals were anesthetized (anesthesia protocol described above) in the dark phase of the light:dark cycle (i.e., when plasma melatonin is elevated). Just before light application, a baseline blood sample was obtained under dim red light illumination (<5 lux). Then, approximately 1500 lux of halogen light (spectrum ∼250–1000 nm with peak between 500 and 900 nm) was applied at eye level for 30 minutes with a blood sample taken at the end of the light application. Maximum sensitivity for melanopsinergic retinal ganglion cells in a diurnal primate has been reported at 482 nm (Dacey et al. 2005). The eyelids were held open and blinked manually every 10 minutes to avoid drying during the light application. Pupillary responses were used to confirm the effectiveness of light placement and were recorded via video recorder. For melatonin stimulation, isoproterenol (ISO; 0.005 mg/bear, i.v.) was administered during the middle of the light phase to four nonanesthetized female bears housed under natural photoperiod in the active season. Blood samples were taken immediately before administration and 3 hours following drug treatment and stored until analyzed for melatonin. This was repeated during hibernation in two anesthetized male bears at the end of their light phase. Given the preliminary findings of unresponsiveness to ISO as well as previous findings in rodents concerning timing of ISO treatment (Steinlechner et al. 1984; Garidou et al. 2002; Kennaway et al. 2002), we delayed the injection of ISO until the end of the light phase. A second ISO injection was then given approximately 1 hour later at twice the active season dose (0.01 mg/bear per injection). Blood samples were collected immediately before ISO administration (baseline) and then 5, 50, 90, and 120 minutes postinjection and assayed for melatonin as described above.

The responsiveness of the hypothalamic–pituitary–adrenal (HPA) axis was evaluated in both active (*n* = 5) and hibernation (*n* = 2) seasons. Only cortisol suppression was determined using the negative feedback of exogenous glucocorticoids on cortisol secretion (Goodman 2009). To this end, betamethasone valerate (BETA; Celestone Soluspan, Schering-Plough, Kenilworth, NJ), a synthetic glucocorticoid, was administered either orally (0.1 mg/kg) in a 50% honey water mixture during the active season or intramuscularly (0.05 mg/kg) during hibernation. Blood samples were collected at approximately 24 and 48 hours following BETA administration and assayed for cortisol as described above.

### Statistical analysis

Hormone data were analyzed using Graphpad Prism5 (La Jolla, CA) and SAS v9.0 statistical software (Cary, N.C.). Because samples were taken at slightly different times of the day across seasons, the values were binned into one of six periods (0100–0300, 0400–0600, 0700–1000, 1100–1500, 1600–1900, and 2000–2400 hours) to create a complete 24-hour profile. Assay values that fell beyond the linear portion of the assay standard curve were replaced with the assay detection limit value. The duration of elevated hormone concentrations was defined as the time between the upward and downward crossing of the daily mean value for each individual (Duffy et al. 2002). Nonanesthetized and anesthetized samples were analyzed separately. Two-way ANOVA or *t*-tests were used to estimate daily and seasonal changes in hormone profiles, variation in duration of hormone secretion, and effectiveness of stimulation or suppression tests. Post hoc analysis was performed using the Bonferroni multiple comparison test. Linear regression analyses were performed on the duration of elevated hormone concentrations relative to the duration of the dark or light phase for each season. Effects were considered statistically significant if *P* ≤ 0.05.

## Results

### Melatonin—nonanesthetized female bears

**Figure 1:**
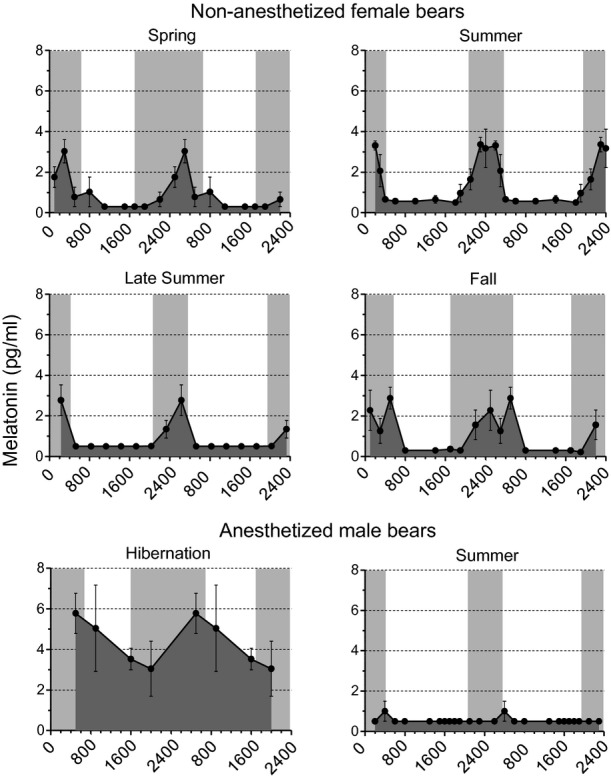
Double-plotted seasonal and daily profiles of peripheral melatonin concentrations (±SEM) under ambient photoperiods for nonanesthetized female and anesthetized male bears (*n* = 4 and *n* = 2–3, respectively). Melatonin varied with time of day in a seasonally dependent manner (*P* < 0.05). Shaded areas represent the dark phase of the L:D cycle. Spring = vernal equinox, March 16–24th; Summer = summer solstice, June 11–27th; Late Summer = August 11–15th; Fall = October 7–16th; Hibernation = December 20th– January 2nd.

**Figure 2:**
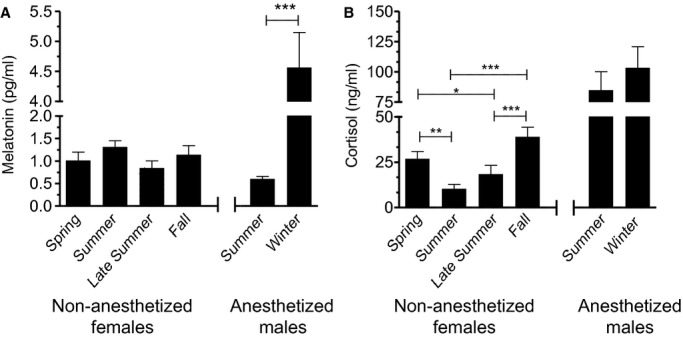
Mean (±SEM) daily concentrations of (A) melatonin (pg/mL) and (B) cortisol (ng/mL). Nonanesthetized and anesthetized groups were analyzed separately. Refer to Figure 1 for sampling date information. *P < 0.05, ***P < 0.001.

Peripheral melatonin concentrations exhibited a significant daily rhythm in all seasons (two-way ANOVA, main effect of time of day, *F* = 12.55, df = 5, *P* < 0.0001; Fig. 1). No main effect of season on mean daily melatonin was measured (*P* = 0.33; Fig. 2); however, an interaction between season and time of day was identified (*F* = 2.53, df = 15, *P* < 0.01). Primarily, this interaction was the result of the significantly higher (*P* < 0.01) concentrations of melatonin during 0400–0600 hours time period in the Fall (2.88 ± 0.46 pg/mL) compared to spring, summer, and late summer (0.64 ± 0.04) (Fig. 1). Additionally, summer values at 2000–2400 hours (2.17 ± 0.23 pg/mL) were higher (*P* < 0.01) than both spring and late summer (avg. 0.80 ± 0.42 pg/mL). The duration of elevated melatonin was significantly related to night length (Fig. 4; Lin. reg; *P* < 0.05, *r*^2^ = 0.28).

### Melatonin—anesthetized male bears

**Figure 3:**
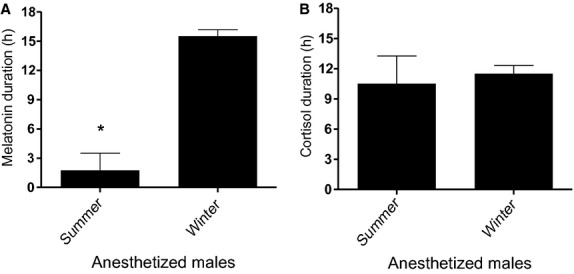
Mean (±SEM) seasonal duration of (A) melatonin and (B) cortisol in anesthetized male bears. Duration defined as the length of time (hour) between the upward and downward crossing of the daily mean concentration. Refer to Figure 1 for sampling date information. *P < 0.05.

**Figure 4:**
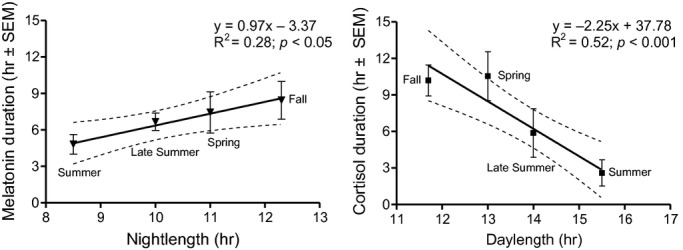
Linear regression (±95% CI) of (A) melatonin duration to night length and (B) cortisol duration to day length for active season (nonanesthetized female bears). Duration defined as the length of time (hour) between the upward and downward crossing of the daily mean concentration. Refer to Figure 1 for sampling date information.

There was no effect of time of day on peripheral melatonin concentrations (two-way ANOVA, *P* = 0.84), but there was a large seasonal difference between mean summer and winter concentrations (Figs. 4; *F* = 28.65, df = 1, *P* < 0.001; 4.35 ± 0.67 vs. 0.55 ± 0.05 pg/mL). Summer anesthetized melatonin levels were just above assay detection limit (assay detection limit = 0.5 pg/mL, summer avg. = 0.55 pg/mL), whereas winter concentrations were tonically elevated (Fig. 1). Additionally, duration of melatonin elevation was much longer in winter compared to summer (Fig. 3; unpaired *t*-test, *P* < 0.01; 15.5 ± 0.66 vs. 1.75 ± 1.75 hours for winter and summer, respectively).

### Cortisol—nonanesthetized female bears

**Figure 5:**
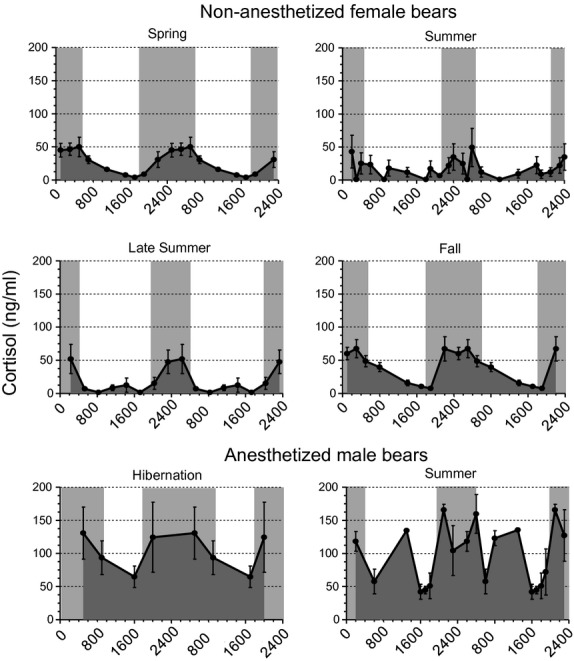
Double-plotted seasonal and daily profiles of peripheral cortisol concentrations (±SEM) for nonanesthetized female and anesthetized male bears (*n* = 4 and *n* = 2–3, respectively). Cortisol varied with time of day in a seasonally dependent manner (*P* < 0.05). Shaded areas represent the dark phase of the LD cycle. Refer to Figure 1 for sampling date information.

Peripheral plasma cortisol concentrations varied with time of day to produce a clear rhythm that also varied with season (Fig. 5; two-way ANOVA, main effect of time of day, *F* = 10.22, df = 5, *P <* 0.0001; and main effect of season, *F* = 7.28, df = 3, *P* < 0.0001). There was no interaction between season and time of day for cortisol concentrations (*P* = 0.13). Cortisol duration was significantly related to changes in day length (Fig. 4; Lin. reg; *P* = 0.001, *r*^2^ = 0.52).

### Cortisol—anesthetized male bears

**Figure 6:**
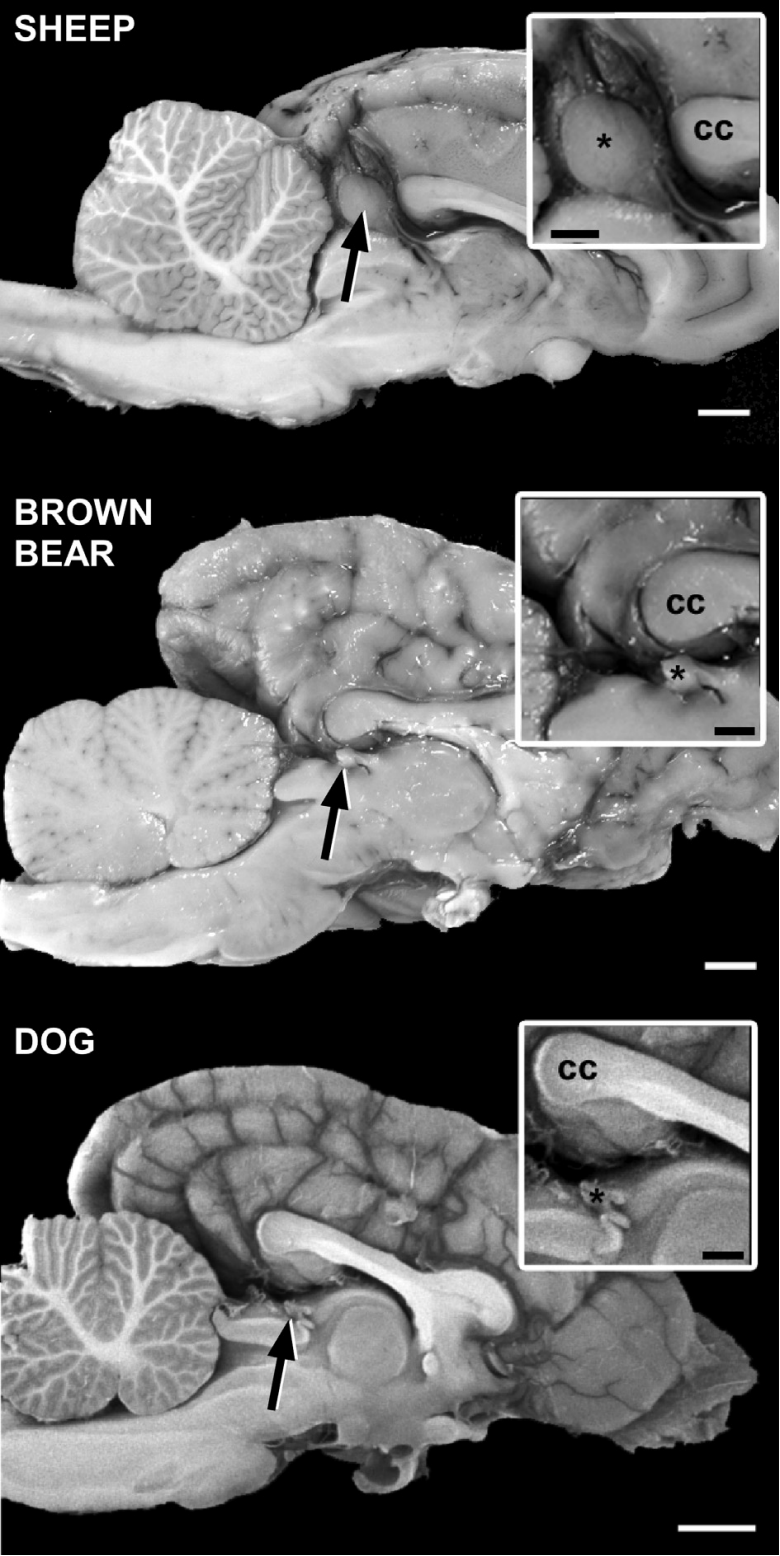
Images of the sheep (Top), brown bear (middle), and dog (bottom) brains illustrating the differences in pineal gland size. Insets represent higher magnification views of the pineal and associated structures. Rostral is to the left in all figures. *cc*, corpus callosum; *pineal gland; Scale bar = 1 cm, inset scale bar = 5 mm.

No effect of either season or time of day on cortisol concentrations was observed in samples taken from anesthetized bears in the summer and winter seasons (Figs. 6; two-way ANOVA, *F* = 1.54, df = 6, *P* = 0.22). The lack of time of day effect was further reflected in a lack of difference in cortisol duration between summer and winter (Fig. 3). In contrast to melatonin, a clear effect of anesthesia on cortisol concentrations was evident (i.e., summer nonanesthetized vs. summer anesthetized values; Fig. 2).

### Pineal gland

**Figure 7:**
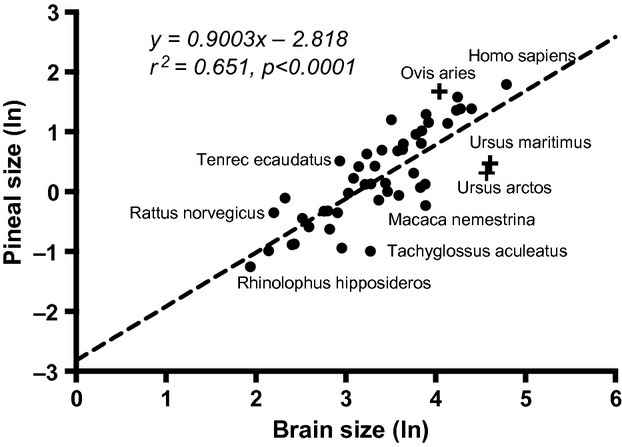
Relationship of the natural log of brain size (width, mm) to natural log of pineal size (width, mm) in 49 different species of mammals. Plus symbols indicate outliers as detailed in Methods. For a list of species analyzed, see Table 1.

An extremely small pineal gland was observed in MRI studies and this was confirmed when the brains were harvested (Fig. 6). The gland size was generally comparable to that of two other related species, the polar bear (not shown) and the dog (Fig. 6). By contrast, bear and dog pineal glands were much smaller than the sheep pineal (Fig. 6). Figure 7 shows pineal size plotted against brain size for 49 different species of mammals using data from the brain museum collection (brainmuseum.org) and the specimens collected for this study (Table 1). Outlier analysis revealed that both bear species and sheep pineal glands, but not the dog pineal, deviated significantly from the expected fit even under the most conservative conditions (Q = 1%). Specifically, pineal size was much smaller than expected in both the brown bear and polar bear and larger in the sheep. Phylogenetic generalized least squares analysis of brain and pineal sizes (width, mm) using the pruned, rescaled phylogeny resulted in a regression line following the form: Pineal = 0.9003 (brain)−2.818

**Table 1:** List of species included in the brain–pineal size analysis

Species	Common name	Species	Common name
*Taxidea taxus*	American badger	*Mandrillus sphinx*	Mandrill
*Castor Canadensis*	American beaver	*Ursus arctos horribilis*^1^	North American brown bear^1^
*Mustela vison*	American mink	*Erethizon dorsatum*	North American porcupine
*Equus burchellii*	Burchell's zebra	*Semnopithecus entellus*	Northern plains gray langur
*Zalophus californianus*	California sea lion	*Thomomys talpoides*	Northern pocket gopher
*Pan troglodytes*	Chimpanzee	*Aotus trivirgatus*	Owl monkey
*Pecari tajacu*	Collared peccary	*Cynocephalus volans*	Phillipine flying lemur
*Sorex araneus*	Common shrew	*Macaca nemestrina*	Pig tailed macaque
*Saimiri sciureus*	Common squirrel monkey	*Ornithorhynchus anatinus*	Platypus
*Tupaia glis*	Common tree shrew	*Ursus maritimus*^1^	Polar bear^1^
*Canis latrans*	Coyote	*Perodicticus potto*	Potto
*Felis silvestris catus*	Domestic cat	*Rattus norvegicus*	Rat
*Canis lupus familiaris*	Domestic dog	*Vulpes vulpes*	Red fox
*Capra hircus domestica*	Domestic goat	*Ailurus fulgens*	Red panda
*Sus scrofa domesticus*	Domestic pig	*Callicebus moloch*	Red-bellied titi
*Ovis aries*	Domestic sheep (Suffolk)	*Macaca mulatta*	Rhesus macaque
*Sciurus carolinensis*	Eastern gray squirrel	*Tachyglossus aculeatus*	Short-beaked echidna
*Scalopus aquaticus*	Eastern mole	*Glaucomys volans*	Southern flying squirrel
*Elephantulus myurus*	Eastern rock elephant shrew	*Crocuta crocuta*	Spotted hyena
*Erinaceus europaeus*	European hedgehog	*Tenrec ecaudatus*	Tailless tenrec
*Homo sapiens*	Human	*Macropus fuliginosus*	Western grey kangaroo
*Pteropus giganteus*	Indian flying squirrel	*Nasua narica*	White nose coati
*Rhinolophus hipposideros*	Lesser horseshoe bat	*Odocoileus virginianus*	Whitetailed deer
*Panthera leo*	Lion	*Bos taurus indicus*	Zebu
*Lama glama*	Llama		

1 Outlier (see Methods for determination of outliers).

(R-squared = 0.651; *P* < 0.0001). There was also a significant correlation between brain size and pineal gland size (T-value = 7.815, *P* < 0.01).

### Melatonin stimulation and suppression tests

**Figure 8:**
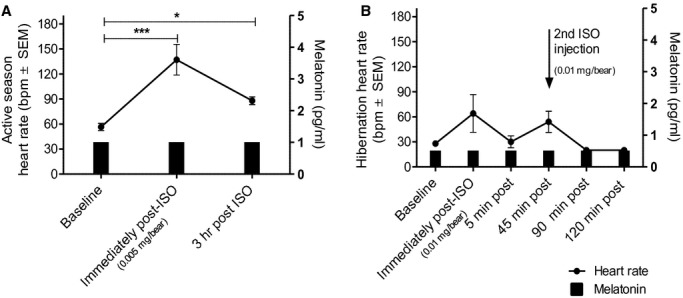
Effects of melatonin stimulation experiments on heart rate (bpm ± SEM) and melatonin in (A) active season, nonanesthetized female bears (*n* = 4, 0.005 mg ISO/bear), and (B) inactive (hibernation) season, anesthetized male bears (*n* = 2; 0.01 mg ISO/bear). There were no effects on melatonin for either season. Arrow indicates time of the second isoproterenol injection; times are relative to time of the first injection. *P < 0.05, ***P < 0.001.

**Figure 9:**
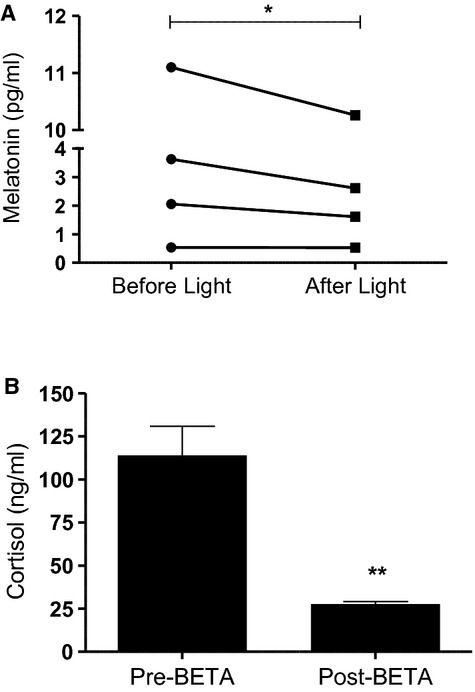
Results of neuroendocrine suppression experiments on (A) melatonin via light application in the inactive season (*n* = 4) and on (B) cortisol via synthetic glucocorticoid (BETA) treatment in both active and inactive season (*n* = 5). *P < 0.05, **P < 0.01.

Regardless of season, dosage, or anesthetic state, ISO failed to cause an increase in peripheral melatonin concentrations; values pre- and post-ISO treatment remained undetectable (<1.0 pg/mL) during the light period (Fig. 8). However, heart rate was significantly elevated following ISO during the active season and remained elevated for 3 hours post-ISO (pre-ISO avg. heart rate = 56.5 ± 4.3 bpm vs. post-ISO avg. heart rate = 137.0 ± 18.4 bpm; Fig. 8A). During hibernation, heart rate increased only transiently after each of the two ISO treatments (Fig. 8B). The hibernation heart rate following ISO was not significantly different from baseline heart rate (unpaired *t*-tests, *P* > 0.05). Bright light application (1500 lux) during hibernation caused a slight, but significant suppression of melatonin by 30 minutes after application (prelight application mean = 4.33 ± 2.3 pg/mL and postlight = 3.75 ± 2.2 pg/mL; Fig. 9A, paired one-tailed *t*-test, *t* = 2.59, df = 3, *P* < 0.05). During the active season, daytime melatonin concentrations were nearly always at the assay detection limit (see Fig. 1), indicative of light suppression, although this was not tested directly.

### Cortisol suppression test

Betamethasone caused a significant and prolonged suppression of plasma cortisol concentrations to below baseline levels for up to 48-hours post administration irrespective of season (Fig. 9B, two-way ANOVA, main effect of treatment, *F* = 12.96, df = 1, *P* < 0.01, main effect of season, *F* = 0.58, df = 1, *P* = 0.46).

## Discussion

Brown bears have evolved a highly seasonal physiology to coincide with annual environmental changes, including food availability. The success of these adaptations presumably relies on the ability to encode a predictable environmental cue such as day length (Goldman 2001; Paul et al. 2008). We reasoned, therefore, that brown bears would express robust seasonal changes in the daily rhythms of two important endocrine hormones, melatonin, and cortisol. Indeed, we found that melatonin and cortisol exhibited clear daily rhythms. However, the seasonal changes in daily rhythms were less obvious. Specifically, no difference in daily mean melatonin concentrations was seen during the early, mid, and late active season. This was somewhat unexpected as seasonal changes in mean daily concentrations of melatonin have been reported for other species (Darrow et al. 1986; Delgado and Vivien-Roels 1989; Edmonds et al. 1995; Vivien-Roels et al. 1997; Goldman 2001; Zawilska et al. 2009). However, given the strong masking effect of light to suppress melatonin, concentrations during the dark phase and the duration of nocturnal elevation should provide a better estimate of seasonal changes in relation to photoperiod. Indeed, we found that the duration of melatonin elevation was significantly affected by changes in night length indicating that the pineal gland in brown bears is responsive to seasonal changes in photoperiod despite the exceedingly low amplitude of the melatonin rhythm and unusually small pineal gland in this species.

In contrast to the low melatonin concentrations observed during the active season, daily melatonin concentrations in hibernation were much greater than those in the summer. Melatonin concentrations were not completely suppressed in the light phase during hibernation, suggesting a baseline increase in melatonin production. The proposed increase is unlikely to be entirely due to the anesthetics because the same bears sedated in the active season (midsummer) had virtually no melatonin detectable at any time of the day. Several other explanations may account for the increased baseline. First, melatonin biosynthetic activity of the bear pineal may be greater during hibernation than other species. This is plausible given the bears’ higher body temperature during hibernation compared to other species in which body temperature is extremely low and melatonin concentrations are greatly reduced (Florant et al. 1984). Second, the metabolic activity of different brain regions varies greatly even in deep torpor as occurs in ground squirrels (Kilduff et al. 1990). The same is likely to be true for the bear. Thus, it is possible that the bear pineal may be more metabolically active relative to other brain regions. Third, reductions in hepatic and renal activity (Hissa 1997) could indirectly slow the clearance of melatonin from the plasma. However, the precise cause of the elevated melatonin during hibernation in the bear remains to be determined.

Because melatonin has both somnogenic (Lerner and Nordlund 1978; Lockley et al. 1997; Zawilska et al. 2009) and hypothermic (Krauchi et al. 1997) properties, the elevated melatonin concentrations we observed during hibernation could provide a mechanism to decrease activity, lower body temperature, and increase the amount of sleep (H.C. Heller, *pers. com*.). Elevated melatonin during hibernation could also have obscured an underlying daily rhythm, consistent with observations made in other hibernators (Florant et al. 1984; Darrow et al. 1986). Indeed, the apparent absence of a (strong) melatonin rhythm is also consistent with reports in other animals living at extreme latitudes where environmental light cycles disappear during the polar summer and winter (Eloranta et al. 1992, 1995; Reierth and Stokkan 1998). However, caution is required in concluding that the melatonin rhythm in bears was completely abolished (vs. masked) because the circadian clock appears functional in bears during hibernation (Ware et al. 2012). A more likely explanation is that either the number of animals was too small or the sampling frequency was insufficient to detect a rhythm.

The absolute concentrations of melatonin measured in bear plasma were exceptionally low compared to other mammals (Arendt 1995; Stehle et al. 2001). Although assay differences could account for some of these differences, comparisons with studies using a similar assay and antibody (Andersson et al. 2000) suggest that this is not entirely the case. More likely, the extremely small pineal gland of the bear relative to its brain size may reflect an anatomical limitation to melatonin production. In support of this hypothesis, adrenergic stimulation failed to increase levels of circulating melatonin in either nonanesthetized female bears during the active season or anesthetized male bears during hibernation, despite heart rate increasing significantly. Furthermore, pineal gland size, but not biosynthetic activity, accounts for a significant amount of the variation in peripheral melatonin concentrations (Coon et al. 1999). Alternatively, an underlying defect in melatonin biosynthesis, similar to what is observed in several mouse strains (Goto et al. 1989) could be a factor, although this seems unlikely given the elevation in plasma melatonin observed during hibernation in our bears. Future studies will be required to differentiate between these possibilities.

Both the polar bear and the brown bear possess pineal glands significantly smaller than expected relative to their brain size. Although a few early studies attempted to define the allometric relationship between pineal size (volume) and body weight, neither ursids nor canids were included in those analyses (Legait et al. 1976a, 1976b). Nevertheless, large differences in pineal size were observed even within mammalian orders leading to the conclusion that pineal gland “must be related to a nonessential somatic function that varies from one species to another at constant somatic weight.” (Legait et al. 1976c). Additionally, phylogenetic correction methods had not been developed at the time which made it difficult to place the findings into a proper phylogenetic context. The present study attempted to overcome these earlier limitations by using modern analytical methods and updated phylogenetic histories. The results of these analyses, after correcting for the influence of phylogeny, reveal that the size of the bear pineal gland is significantly smaller than that of other mammals. Clearly, brown bears and polar bears have been successful even with a relatively small pineal gland and very low circulating melatonin concentrations.

A functioning pineal gland is necessary for seasonal responses to photoperiod (Goldman 2001). Based on the light suppression (albeit slight) that we observed during hibernation in combination with the very low levels during the day in the active season, bear melatonin is capable of responding to photic signals. In contrast to other mammals, including a diurnal rodent, the grass rat (*Arvicanthis ansorgei*) (Romero and Axelrod 1975; Garidou et al. 2002; Kennaway et al. 2002), bears were not responsive to β-adrenergic stimulation of melatonin during the light phase despite producing an increased heart rate (during the active season). This may be indicative of a nighttime ‘gating’ effect as seen in several hamster species (Reiter et al. 1987; Hong et al. 1993; Garidou et al. 2003; Simonneaux and Ribelayga 2003). Future studies are required to confirm this in bears.

Daily cortisol profiles in the bears were very similar to those described for other diurnal species suggesting a diurnal niche for the brown bear as we hypothesized previously (Ware et al. 2012). Unlike melatonin, mean daily concentrations of cortisol varied significantly with season. Cortisol elevations were also strongly dependent on day length. Seasonal changes in cortisol are well documented (Saboureau et al. 1979; Boswell et al. 1994; Gower et al. 1996; Gardiner and Hall 1997) with greater concentrations of cortisol associated with periods of food scarcity (Bubenik et al. 1983; Palumbo et al. 1983; Harlow and Beck 1990; Saltz and White 1991; Tsuma et al. 1996; Huber et al. 2003). The effects of cortisol are generally to mobilize energy reserves via lipolysis (Hadley 1984; Granner 1985). Increased use of lipids during prolonged fasting and hibernation has been extensively documented in various birds and mammals (Allen 1976; Young 1976; Geiser and Kenagy 1987). This action, occurring primarily on fat reserves, protects valuable lean (muscle) tissue (Granner 1985). On the basis of these earlier findings, we predicted that bears would have higher levels of cortisol during hibernation compared to the active season. Unfortunately, we could not confirm our prediction because of the effect of the anesthetics to increase basal cortisol. Despite the lack of a statistically significant change in cortisol during the winter in anesthetized male bears, the gross rhythm, with appropriate phasing (i.e., elevated in the dark phase and declining during the light phase) was maintained. Because we could not sample unanesthetized bears during hibernation, additional studies will be required to address this. A baseline elevation in cortisol during hibernation would, however, be consistent with its role in facilitating lipolysis and energy metabolism, as previously suggested for bears (Palumbo et al. 1983; Harlow and Beck 1990; Hellgren et al. 1993). Previous studies in brown bears examining plasma cortisol have only been conducted during the active season and under anesthesia (Brannon 1985; Cattet et al. 2003). In those studies, cortisol concentrations ranged from 54 to 177 ng/mL (Brannon 1985; Cattet et al. 2003), very similar to our active season anesthetized average of 85 ng/mL. Intriguingly, we observed that in nonanesthetized female bears, adults had undetectable levels of cortisol during midsummer compared to subadults. Although the small sample size prevented us from statistically comparing these age groups, both age and reproductive status are reported to affect cortisol concentrations (Harlow and Beck 1990; Coe and Levine 1995; Kiess et al. 1995; Schiml et al. 1996; Ruis et al. 1997; Boonstra et al. 2001; McKenzie and Deane 2003), though some reports found no effects of age (Huber et al. 2003; Christina et al. 2004). Sex of the bears may also have impacted results between seasons. The nonanesthetized samples were collected exclusively from female bears while the anesthetized samples were obtained only from males. Generally, males have been found to have higher basal levels of circulating cortisol than females (Ruis et al. 1997). Circulating binding factors, such as corticosterone-binding globulin (CBG), exhibit sexual dimorphism with adult females having elevated CBG, thus potentially lowering plasma cortisol concentrations (Allen 1976; Chow et al. 2010).

The very low cortisol levels in adult nonanesthetized bears during the summer were in sharp contrast to those measured in anesthetized bears during the same period confirming that the anesthetics used in our studies caused the increases. Indeed, sympathomimetics, such as that used in the current studies have been documented to increase cortisol (Al-Damluji et al. 1987; Bugajski et al. 1991; Lin et al. 1993; Clapper 2008). In contrast, the benzodiazepine-like drug, Zolazepam, included in Telazol, could mitigate some of the HPA stimulation resulting from tiletamine hydrochloride (Rohrer et al. 1994; Imaki et al. 1995; Bentson et al. 2003). Lastly, medetomidine was also included in our anesthetic cocktail and has been reported to reduce sympathetic activation (Aho et al. 1992; Caulkett et al. 1999; Ko et al. 2000). The most parsimonious explanation of the elevated cortisol is that the repeated doses of Telazol were responsible for continued elevation in basal cortisol. It is interesting that melatonin was not affected in a similar way, although the α-2 adrenergic agonist dexmedetomidine may have played a role (Mustanoja et al. 1997).

### Perspectives and Significance

Melatonin and cortisol both appear to serve as seasonal phase markers in the brown bear. The nocturnal elevation in melatonin in brown bears is consistent with observations made in many other temperate species. However, the extremely small amplitude observed may be related to the unexpectedly small pineal gland in the bear. We hypothesize the very low nocturnal melatonin concentrations during the active season may facilitate a certain degree of behavioral flexibility allowing brown bears to adapt to highly variable food resources and diverse temporal niches (Ware et al. 2012). The elevated baseline melatonin during hibernation may be a unique adaptation in this large mammal to facilitate metabolic suppression by lowering body temperature and enhancing sleep propensity. The daily cortisol profile is very similar to that of other diurnal species suggesting that this is the preferred temporal niche of the brown bear.
